# Multimodal strategies to hand hygiene in Ghanaian hospitals: a cross-sectional study in the Eastern Region of Ghana

**DOI:** 10.1136/bmjph-2023-000606

**Published:** 2024-02-05

**Authors:** Stephen Dajaan Dubik, Kingsly E Amegah, Ama Akyampomaa Owusu-Asare, Akosua Takyiwa Kwakye, Christiana Akufo, Joyce Amponsah, Hectoria Awekeya, Leslie Vander Puije, Jocelyn Asibey, Seth Twum, Francis Mensa Akwetey, Portia Sam, Winfred Ofosu, Angela Ackon, Sofonias Asrat, Hedidor George Kwesi, Sally-Ann Ohene, Mary Eyram Ashinyo

**Affiliations:** 1 School of Allied Health Sciences, University for Development Studies, Tamale, Ghana; 2 Department of Data Science and Economic Policy, University of Cape Coast, Cape Coast, Ghana; 3 World Health Organization Country Office, Accra, Ghana; 4 Department of Quality Assurance, Ghana Health Service, Accra, Ghana; 5 Eastern Regional Health Directorate, Ghana Health Service, Accra, Ghana; 6 World Health Organization Country Office Ghana, Accra, Ghana; 7 Ghana Health Service, Ghana Ministry of Health, Accra, Ghana; 8 Maternal and Child Health, University of North Carolina at Chapel Hill Gillings School of Global Public Health, Chapel Hill, North Carolina, USA

**Keywords:** Hand Hygiene, Hygiene, Public Health

## Abstract

**Background:**

Hand hygiene (HH) is one of the core components of infection prevention and control and is critical for a high quality of care. Multimodal approaches are recommended to strengthen and drive HH systems in healthcare facilities (HCFs). We aimed to assess the extent of implementation of the WHO HH multimodal improvement strategy in HCFs in the Eastern Region of Ghana.

**Methods:**

This study was a descriptive cross-sectional study involving 22 HCFs from 17 districts in the Eastern Region of Ghana. We collected data from 22 hospitals using the WHO Hand Hygiene Self-Assessment Framework (HHSAF). Data were analysed through descriptive statistics.

**Results:**

The HHSAF median score denotes an intermediate HH implementation level (53.5%, IQR 48.8%–58.3%). Fourteen HCFs attained an intermediate level, five attained basic level, one attained advanced level and no facility exhibited inadequate HH implementation level. Evaluation and feedback had the highest score (64.3%, IQR 50%–71.4%), as ward-based audits for the availability of HH resources have become standard practice in many of the HCFs. Reminders in the workplace had the lowest score (33.3%, IQR 25.9%–37.0%), whereby less than half (46%) of the HCFs had posters explaining the indications for HH and the correct use of alcohol-based hand rubs. HH implementation level did not differ significantly among government (M=49.97, SD=12.30) and non-government (M=53.32, SD=18.73) facilities, (t (20)=−0.503, p=0.621).

**Conclusion:**

Most HCFs had an intermediate HH implementation level. The provision of HH resources, including posters, HH rounds in patient care areas, introduction of HH corners, leadership, financial and organisational support are key elements for increased compliance with the WHO HH multimodal improvement strategy.

**WHAT IS ALREADY KNOWN ON THIS TOPIC**

**WHAT THIS STUDY ADDS**

- This Hand Hygiene Self-Assessment Framework survey showed most healthcare facilities (HCFs) in the Eastern Region of Ghana had an intermediate HH implementation level. Most of the HCFs excelled in evaluation and feedback, institutional safety climate, and education and training, but performed poorly in system change and reminders in the workplace.

**HOW THIS STUDY MIGHT AFFECT RESEARCH, PRACTICE OR POLICY**

- The provision of HH resources, including posters, HH rounds in patient care areas, introduction of HH corners, leadership, financial and organisational support are key elements for increased compliance with the WHO HH multimodal improvement strategy.

## Introduction

 Hand hygiene (HH) is one of the core components of infection prevention and control (IPC) in healthcare facilities (HCFs) and is critical for high quality of care and patient safety. Multimodal approaches are recommended to strengthen and drive HH systems in HCFs to prevent healthcare-associated infections (HAIs). HAIs are a major public health concern, affecting healthcare quality and patient safety across the globe. For instance, a study by Jha *et al* showed that globally, 421 million annual hospitalisations translate to approximately 43 million harms to patients during healthcare delivery worldwide.^1^ HAIs are deemed most significant in developing countries, resulting in 2.6 million deaths.^2^ Additionally, HAIs affect 15.5% of patients each year, and antimicrobial resistance (AMR) alone leads to 700 000 mortalities in developing countries.^3^ In the USA, it was estimated in 2009 that HAIs incidence in hospitals ranged from 1.7 to 23.6 per 100 admitted patients, translating to a yearly hospital cost of US$28.4–US$33.8 billion^4^ and 98 987 deaths per year.^5^

Based on the data above, it is clear that HAIs result in avoidable deaths, exert economic burden, prolonged hospital stay, create disability and increase the burden of AMR.^6^ For example, estimates by the WHO showed that 56% of neonatal deaths are due to HAIs,^6^ yet, HAIs are efficiently prevented through IPC protocols, including HH. For instance, some scholars suggest that improved compliance with HH can reduce HAI rate by 40%^7^ and the risk of acquiring methicillin-resistant *Staphylococcus aureus*.^8^ The role of HH in preventing infections informs the basis for the WHO recommendations that all healthcare workers must wash their hands at critical moments, namely, before patient contact, before an aseptic task, after body fluid exposure risk, after patient contact and after contact with patient surroundings.^9^ Despite this recommendation, 61% of health workers across the globe do not adhere to appropriate HH practices during healthcare.^4^ Also, a survey in 54 low-income and middle-income countries showed that 35% of the HCFs do not have water and soap for handwashing.^10^

In Ghana, adherence to HH among healthcare workers is low despite known benefits to both health workers and patients.^11 12^ Globally, many factors have contributed to the poor adherence to HH among health workers. These include lack of training, inadequacy or lack of clean water in healthcare settings, poor feedback mechanisms, work overload, inadequate staff, poor structuring of workplaces, inadequate alcohol-based cleaning agents, as well poor administrative commitment to HH.^13 14^ The WHO launched the world HH day in 2009 to help bring people together to support healthcare workers to improve HH in healthcare. It was part of a global patient safety strategy by the WHO under the broad theme ‘SAVE LIVES: Clean Your Hands’ campaign geared towards safe care. In Ghana, the 2022 National Global Handwashing Day was held in the Eastern region of Ghana. In commemoration of this and to understand the status of HH implementation in the HCFs, we conducted a rapid survey of HH implementation tactics using the WHO multimodal HH improvement strategy. It is well documented that the WHO multimodal HH improvement strategy is sustainable and effective in increasing HH compliance, especially in resource limited settings such as Ghana.^15^

## Materials and methods

### Study settings

The Eastern Region is situated in the Southern part of Ghana. It has 33 Metropolitan, Municipal and Districts Assemblies (MMDAs). The Region covers 19 323 km^2^ and shares borders with Lake Volta to the East, Bono Region and Ashanti Region to the North, Ashanti Region to the West, Central and Greater Accra Regions to the South. The Eastern Regional capital town is Koforidua, and key ethnic groups are Akans, Krobos and Ewes.^16^ Through the support of the WHO country office in Ghana, the Ghana Health Service commissioned this study in commemoration of the 2022 Global Handwashing Day in the Eastern region of Ghana. A total of 15 (68%) government and 7 (32%) private facilities participated in the survey. The study period lasted from 1 April 2022 to 30 April 2022.

### Study design

The study was a descriptive cross-sectional study involving 22 HCFs in the Eastern Region of Ghana.

### Survey instrument, measurement and data collection

The data were collected using the WHO Hand Hygiene Self-Assessment Framework (HHSAF). The HHSAF is a tool used to conduct a situational analysis of HH promotion and practices within an HCF. The HHSAF presents an opportunity for actors in the healthcare industry to reflect on existing resources and achievements in HH and shape future plans and challenges. Additionally, the HHSAF serves as a diagnostic tool for identifying key issues about HH that require attention and improvement.^17^

The HHSAF tool has five multimodal components: system change, education and training, evaluation and feedback, reminders in the workplace, and institutional safety climate. The study tool comprised 47 questions. Overall, 6 questions measured indicators for system change, 8 questions for education and training, 10 questions for evaluation and feedback, 7 questions for reminders in the workplace and 16 questions for institutional safety climate. The overall attainable HHSAF score of 635 was converted into percentages and classified to reflect the HCFs performance based on the WHO HHSAF levels. Based on the score, an HCF was categorised on HH implementation level as follows: inadequate (0%–25%), basic (26%–50%), intermediate (51%–75%) and advanced (76%–100%). The meaning of inadequate, basic, intermediate and advanced levels is explained in table 1.

**Table 1:** Levels of hand hygiene implementation and its interpretation by the WHO^17^

Hand hygiene implementation level	Meaning
Inadequate	Hand hygiene practices and hand hygiene promotion are deficient. Significant improvement is required
Basic	Some measures are in place but not to a satisfactory standard. Further improvement is required
Intermediate	An appropriate hand hygiene promotion strategy is in place, and hand hygiene practices have improved. It is now crucial to develop long-term plans to ensure that improvement is sustained and progresses.
Advanced	Hand hygiene promotion and optimal hand hygiene practices have been sustained and/or improved, helping to embed a culture of safety in the healthcare setting.

### Sampling

We purposively invited the IPC focal persons in each hospital to collect data at the hospital level. All 44 hospitals (comprising both public and private hospitals) in the Eastern region were invited to participate in the study. We analysed data for only 22 hospitals due to non-response from the remaining hospitals. Hospitals that did not give feedback after 1 week of the distribution were consider non-participants. This was necessary to allow for the celebration of the National Hand Hygiene Day. The survey was conducted because of the celebration of National Hand Hygiene Day in the Eastern region, and the findings of the survey were scheduled to be shared with stakeholders on Hand Hygiene Day in Ghana. Before data collection, the IPC focal persons were trained virtually on the HHSAF tool. Data collection period lasted for 7 days.

### Data analysis

The data were analysed using STATA V.16. The data were downloaded in an Excel format, cleaned and exported to STATA for further analysis. The data were summarised using median, frequency and percentages. An independent sample t-test was conducted to determine the differences between the scores of the five HHSAF components.

## Results

### The overall HH implementation levels in the HCFs

Twenty-two HCFs from 17 districts in the Eastern Region participated in the survey. The participating hospitals included 6 mission hospitals, 1 private hospital and 15 government hospitals. The HHSAF score denotes an intermediate HH implementation level in the HCFs (53.5%, IQR 48.8%–58.3%). The HHSAF scores for the various components were system change 57.1% (IQR 42.9%–57.1%); education and training 59.5% (IQR 47.6%–66.7%); evaluation and feedback 64.3% (IQR 50%–71.4%); reminders in the workplace 33.3% (IQR 25.9%–37.0%) and institutional safety climate 60.0% (IQR 46.7%–66.7%), which all reflect an intermediate level (table 2).

**Table 2:** Hand hygiene multimodal strategy implementation levels in the healthcare facilities

Facility code	SC	ET	EF	RW	ISC	HHL
F1	57.1	66.7	71.4	33.3	73.3	60.6
F2	42.9	57.1	78.6	25.9	60.0	53.5
F3	66.7	52.4	57.1	33.3	46.7	50.4
F4	47.6	23.8	60.7	14.8	46.7	39.4
F5	47.6	76.2	39.3	40.7	46.7	48.8
F6	57.1	66.7	57.1	33.3	66.7	55.9
F7	57.1	66.7	71.4	37.0	40.0	53.5
F8	57.1	4.8	7.1	7.4	13.3	16.5
F9	66.7	47.6	71.4	25.9	60.0	54.3
F10	52.4	61.9	35.7	44.4	53.3	48.8
F11	66.7	76.2	71.4	37.0	80.0	66.1
F12	38.1	23.8	10.7	11.1	6.7	16.5
F13	66.7	76.2	78.6	33.3	80.0	66.9
F14	66.7	52.4	64.3	33.3	66.7	56.7
F15	57.1	52.4	64.3	37.0	53.3	52.8
F16	33.3	38.1	50.0	11.1	33.3	33.1
F17	42.9	66.7	71.4	37.0	53.3	54.3
F18	57.1	85.7	78.6	63.0	93.3	76.4
F19	38.1	66.7	78.6	37.0	66.7	58.3
F20	47.6	42.9	67.9	25.9	60.0	49.6
F21	57.1	57.1	64.3	33.3	80.0	59.1
F22	38.1	66.7	42.9	48.1	60.0	51.2
Overall score(Median: IQR)	57.1†(42.9–57.1)	59.5†(47.6–66.7)	64.3*‡(50.0–71.4)	33.3*(25.9–37.0)	60.0†(46.7–66.7)	53.5†(48.8 58.3)

* Basic.

† Intermediate.

‡ Advanced.

EF, evaluation and feedback; ET, education and training; HHL, hand hygiene levelISC, institutional safety and climate; RW, reminders in the workplace; SC, system change

### System change

The analysis showed that 68% of HCFs surveyed reported the availability of a facility-wide continuous supply of alcohol-based hand rub at each point of care. Most facilities (72.7%) had at least a 1:1 HH station to bed ratio in their isolation rooms and intensive care units and almost all (96%) reported a continuous supply of clean running water in their facility. Similarly, soap was always available in 91% of the reported HH stations in the health facilities, 73% of the HCFs had single-use towels available at each HH station and about 96% of the HCFs management were committed to providing alcohol-based hand-rub and soap for use at all times (online supplemental file 1).

### Education and training

While training on HH for healthcare professionals was done annually in 36% of the HCFs surveyed, 55% of the facilities had processes to confirm whether healthcare workers completed HH training. Most facilities (68%) had documents on ‘the 5 Moments for Hand Hygiene’ in healthcare, 96% had documents on ‘Steps in Hand Hygiene’ and more than half (63.6%) had documents on ‘National IPC/WASH guidelines’. Almost all (n=20 900.9%) health facilities had active IPC focal persons with adequate skills who served as trainers for HH educational programmes. Systems were in place in 14 (63.6%) health facilities to check HH compliance among healthcare workers. Less than half (41%) of the facilities had a dedicated budget for IPC training, including HH (online supplemental file 1).

### Evaluation and feedback

Ward-based audits were conducted to assess the availability of HH resources. About 86% of health workers had knowledge of the indication for HH and 91% had knowledge on the correct technique for HH. Thirteen (59%) of the HCFs monitored the consumption of alcohol-based hand rub and 15 (68%) monitored soap consumption at the health facilities at least every 3 months. Nine (41%) of the HCFs directly observed staff HH compliance using the five moments for HH. Another 10 (46%) of the HCFs used the HH steps in the National IPC Guidelines to assess improvement every 3 months in the health facilities. Immediate coaching and mentorship were provided for most (77%) frontline healthcare workers during HH compliance assessment in the facilities. Most (n=18, 81.8%) of the health facilities gave feedback information on HH compliance to healthcare workers and facility leadership or management (online supplemental file 1).

### Reminders in the workplace

Less than half, (46%) of the HCFs had posters explaining the indications for HH and the correct use of alcohol-based hand rub. A poster explaining hand washing techniques were reported to be displayed at every sink in the wards and treatment areas in 50.0% of the HCFs. Most (n=14, 64%) of the health facilities could not undertake HH promotional activities by displaying and regularly updating posters. HH information and other workplace reminders such as leaflets, HH campaign screen savers, stickers and badges were unavailable in 19 (86.4%) of all the wards throughout the health facilities (online supplemental file 1).

### Institutional safety climate for HH

An IPC/WASH committee was available in 73% of the HCFs, and 50.0% of these facilities had dedicated time to conduct active HH promotions. HCFs reported the commitment of front-line managers such as health service administrators (77%), medical superintendents (91%) and deputy directors of nursing service (91%) to HH improvement programmes. Only one HCF reported celebrating the Global Hand Hygiene Day in the last 2 years. Few (23%) of the HCFs had systems to designate HH champions, and most (91%) of the HCFs did not have systems for recognition of HH models. HH education for patients was conducted in 96% of all the HCFs. Eighty-six per cent of the HCFs lacked initiatives to support HH e-learning, 59% had IPC/HH included in staff appraisal, and student interns, and new employee orientation on HH was done in 91% of the HCFs (online supplemental file 1).

### HHSAF assessment scores of the 22 HCFs in the Eastern Region of Ghana

On system change, more than half (59%) of the hospitals were on the intermediate level of HH implementation, while less than half (41%) of the hospitals rated basic level in HH implementation. Education and training marker had 12 (55%) of the hospitals attaining scores reflecting an intermediate level of HH hygiene implementation, 18% of the hospitals attained advanced level and 14% had scores reflecting an inadequate level of HH implementation (table 3).

**Table 3:** Hand hygiene implementation level according to healthcare facility status

HHSAF components	Hospital status		
	Governmental hospital	Non-governmental	All facilities
	N=15	N=7	N=22
System change			
Basic level	7 (46.7)	2 (28.6)	9 (40.9)
Intermediate level	8 (53.3)	5 (71.4)	13 (59.1)
Training and education			
Inadequate	2 (13.3)	1 (14.3)	3 (13.6)
Basic level	3 (20.0)	0 (0.0)	3 (13.6)
Intermediate level	8 (53.3)	4 (57.1)	12 (54.5)
Advanced level	2 (13.3)	2 (28.6)	4 (18.2)
Evaluation and feedback			
Inadequate	1 (6.7)	1 (14.3)	2 (9.1)
Basic level	2 (13.3)	2 (28.6)	4 (18.2)
Intermediate level	10 (66.7)	2 (28.6)	12 (54.5)
Advanced level	2 (13.3)	2 (28.6)	4 (18.2)
Reminder in the workplace			
Inadequate	3 (20.0)	1 (14.3)	4 (18.2)
Basic level	12 (80.0)	5 (71.4)	17 (77.3)
Intermediate level	0 (0.0)	1 (14.3)	1 (4.5)
Institutional safety climate			
Inadequate	1 (6.7)	1 (14.3)	2 (9.1)
Basic level	5 (33.3)	0 (0.0)	5 (22.7)
Intermediate level	7 (46.7)	4 (57.1)	11 (50.0)
Advanced level	2 (13.3)	2 (28.6)	4 (18.2)
Overall hand hygiene level			
Inadequate	1 (6.7)	1 (14.3)	2 (9.1)
Basic level	5 (33.3)	1 (14.3)	6 (27.3)
Intermediate level	9 (60.0)	4 (57.1)	13 (59.1)
Advanced level	0 (0.0)	1 (14.3)	1 (4.5)

HHSAFHand Hygiene Self-Assessment Framework

The evaluation and feedback domain had the majority (55%) of the hospital attaining scores that reflect intermediate level, 18% reached an advanced level and another 18% had scores ranging from 35.7% to 50.0%, reflecting basic level. The hospitals performed poorly on reminders in the workplace domain. Thirty-two per cent of the hospitals had scores ranging from 7.4% to 25.9%, reflecting inadequate HH implementation, 64% were classified as basic level and only one facility attained intermediate level. On institutional safety climate for HH, 23% of the hospitals had scores ranging from 33.3% to 46.7%, denoting basic level, half of the hospitals had scores ranging between 53.3% and 73.3%, reflecting intermediate level and 14% were classified as advanced level (table 3).

The overall performance of the HCF on the HHSAF revealed that the majority (n=14, 63.6%) were at the intermediate level, 5 (22.7%) were at basic level and 2 (9.1%) were at inadequate level. Only 1 (4.5%) HCFs attained the advanced level of HH (table 3).

### Differences in HHSAF scores according to HCF status

We found no statistically significant difference in all the five HHSAF component scores between both government and non-governmental HCFs (p>0.05). Overall, HH level did not differ significantly among government (M=49.97, SD=12.30) and non-government (M=53.32, SD=18.73) HCFs, (t (20)=−0.503, p=0.621) (table 4).

**Table 4:** Bivariate analysis of differences in HHSAF scores according to healthcare facility status

HHSAF components	Government hospital	Non-government hospital	Difference	P value
	Mean (SD)	Mean (SD)	Mean (difference)	
System change	53.02 (11.08)	52.38 (10.65)	0.63	0.901
Education and training	53.3 (16.32)	61.22 (26.14)	7.89	0.394
Evaluation and feedback	61.43 (17.45)	53.06 (26.36)	8.37	0.384
Reminders in the workplace	28.89 (9.62)	38.62 (16.41)	9.74	0.099
Institutional safety climate	53.78 (18.93)	61.90 (25.16)	8.13	0.408
Total score	49.97 (12.30)	53.32 (18.73)	3.34	0.621

HHSAFHand Hygiene Self-Assessment Framework

## Discussion

To the best of our knowledge, this is the first study in Ghana that has assessed the implementation of multimodal HH improvement strategy using the WHO HHSAF tool. The findings of this study have implications for IPC policies, programming and healthcare quality in Ghana. Our findings showed that hospitals in Ghana’s Eastern Region had overall multimodal HH score of 53.5% (IQR 48.8%–58.3%), reflecting an intermediate level of HH implementation. This is in line with a similar HHSAF assessment in the Republic of Sierra Leone, where the implementation of HH in 13 public hospitals was mainly at an intermediate level.^18^ On the contrary, a baseline survey in the Dodoma Region in Tanzania found that most hospitals were rated inadequate HH implementation level.^19^ The overall HHSAF score in this study is lower than what was reported by a global survey in 2019^20^ and in the USA.^21^ The implementation of appropriate HH structures in developing countries has been constrained by barriers such as lack of HH resources, heavy workload, blocked/leaking sinks and poorly positioned facilities.^22^ This may be the reason for the intermediate level of HH implementation among the hospitals studied.

Contrary to the findings in Cambodia where no facility achieved intermediate level,^23^ most of the facilities in this survey attained an intermediate level of HH implementation. In Italy, most (70.4%) HCFs were rated intermediate level^24^ than was observed in this survey. Ghana has developed and implemented national guidelines on IPC in HCFs for close to two decades now^25^; perhaps, this may be the possible explanation for the intermediate level performance of the HCFs compared with other developing countries. Only one hospital attained an advanced level; in Sierra and Cambodia, no hospital achieved an advanced level performance.^18 23^ This showed that more coordinated efforts are needed to sustain the gains and propel the HCFs to advanced level.

The highest scored element was evaluation and feedback, where ward-based audits for the availability of HH resources, healthcare worker knowledge of the indication of HH and correct techniques for HH were done in many of the hospitals. Unlike other surveys in similar low-income and middle-income countries, where the lowest scoring HHSAF elements were evaluation and feedback,^20^ and institutional safety climate,^18 20^ in this study, we observed lower scores among reminders in the workplace. These variations may be due to discrepancies in the health systems and IPC challenges unique to each country. Contrary to findings from other scholars,^18 23^ the HHSAF lowest scoring element was reminders in the workplace. This finding may be an indication of the lack of resources to procure HH materials such as posters. A major gap in the reminders in the workplace was the lack of posters explaining the indications for HH and correct use of alcohol-based hand rub, lack of HH promotion using posters and unavailability of leaflets, HH campaign screen, stickers and badges. Previous study by scholars in the USA suggest that posters play a role in improving HH compliance.^26^ Hence, the unavailability of these HH reminders in the HCFs may affect HH practice at critical moments, thereby increasing HAI rates among healthcare workers. Our findings highlight the need for better financial support to the HCFs to enable proper implementation of the WHO multimodal HH improvement strategy.

The analysis of the system change element showed that almost all the HCFs surveyed reported a continuous supply of clean running water, availability of soap at HH station and management commitment to the provision of alcohol-based hand rub. These findings are not surprising as the outbreak and response to the COVID-19 pandemic might have strengthened water, sanitation and hygiene issues in HCFs.^27^ Despite these gains, there were gaps in the provision of single-use hand towels in the HCFs. This is also the case with similar HHSAF assessments in developing countries.^18 23^ The inadequacy of the HH resources may be because most of the HCFs did not have a dedicated budget for IPC-related activities, including HH. Therefore, the Government of Ghana and its development partners should intensify the provision of funds and HH resources to HCFs in Ghana. The role of HH in minimising HAIs and improving treatment outcomes^28^ could be derailed if the HCFs do not have adequate supply of HH resources.

For education and training, a major gap was the availability of a dedicated budget for training of healthcare workers on IPC. Inadequate budgets for IPC-related activities have been emphasised in Ghana^29^ and the Republic of Sierra Leone.^30^ The unavailability of budget for IPC activities and training could affect optimal IPC practices, HAI surveillance and monitoring, and audit of IPC practices. Almost all the facilities had active IPC focal persons with adequate skills who served as trainers for HH educational programmes. Compared with our findings, an assessment in 14 low-income and middle-income countries showed the availability of IPC/water, sanitation and hygiene (WASH) focal persons was suboptimal. IPC focal persons play a critical role in HAI surveillance, serving as trainers for educational programmes and monitoring of HH compliance among peers.^31^ Education and training is the cornerstone for HH compliance, and it has been shown to be effective in promoting HH.^32^

The major gaps in the institutional safety climate were the unavailability of designated HH champions, lack of dedicated time to conduct HH promotion activities and lack of inclusion of HH in staff appraisal. This key deficiency has been emphasised in similar assessments.^18 20 23^ An institutional safety climate is crucial in building HH leadership, continuing HH activities and motivating HH compliance among healthcare workers.^17^ The overall score on institutional safety climate is better than previously published studies.^18 24^ We did not find significant differences between government and non-government facilities. We anticipated this finding because most of the non-government facilities were mission facilities. Mission facilities, such as government facilities, are supported by the Government of Ghana in the form of human resources, capacity building and infrastructure.

### Strengths and limitations of the study

The survey was self-administered by the IPC focal persons. We could not independently verify the information provided by the IPC focal persons. Therefore, we acknowledge that the findings may be constrained by potential social desirability bias. However, we do not doubt the competencies of the IPC focal persons to provide accurate data that depict the HH implementation level in the HCFs. The selected HCFs were hospitals; hence the findings of this study cannot be generalised in relation to HCFs that are non-hospitals. We invited the HCFs to participate voluntarily, which means facilities that were more compliant were likely to complete the survey. This may render our findings to potential bias and may skew the results towards high performance. The study was conducted in the Eastern Region of Ghana, we want to caution readers from generalising our findings to other regions or countries. However, the participating hospitals were drawn across 17 MMDAs in the Eastern Region of Ghana. This renders our study results representative in the regional context. Additionally, our study included government, mission and private HCFs.

## Conclusion

This HHSAF survey showed most HCFs in the Eastern Region of Ghana had an intermediate HH implementation level. There were variations in performance across the domains, with most of the HCFs excelling in evaluation and feedback, institutional safety climate, and education and training. The system change element and reminders in the workplace are key areas that are difficult to implement by the HCFs. The provision of HH resources, including posters, HH rounds in patient care areas, introduction of HH corners, leadership, financial and organisational support are key elements for increased compliance with the WHO HH multimodal improvement strategy. Additionally, it is crucial for the HCFs to develop long-term plans to ensure that the HH improvement is sustained and progresses to the advanced level of HH implementation. For the facilities to progress to the advance level of implementation, multiple barriers to HH such as weak institutional support, inadequate HH materials, heavy workloads, inadequate HH training and poorly designed wards should be addressed.

## supplementary material

10.1136/bmjph-2023-000606 online supplemental file 1

## Data Availability

All data relevant to the study are included in the article or uploaded as online supplemental information.
