# Supplementary material for: Multimodal strategies to hand hygiene in Ghanaian hospitals: a cross-sectional study in the Eastern Region of Ghana
Source: BMJ Public Health. 2024 Feb 5;2(1):e000606. doi: 10.1136/bmjph-2023-000606 (PMC11816844; doi:10.1136/bmjph-2023-000606)
Supplement: online supplemental file 1 [file bmjph-2-1-s001.pdf]

Descriptive analysis of the various components of the WHO hand hygiene self-assessment framework (HHSAF).

Table 1: System Change

System Change	Frequency	Per cent %
Availability of alcohol-based hand rub in the healthcare facility		
Available only in some wards or in discontinuous supply	1	4.5
Available facility-wide with a continuous supply	3	13.6
Available facility-wide with continuous supply and at the point of care in the majority of wards	3	13.6
Available facility-wide with continuous supply at each point of care	15	68.2
Hand hygiene station to bed ratio		
Less than 1:10	6	27.3
At least 1:10 facility-wide and 1:1 in isolation rooms and intensive care units	16	72.7
Continuous supply of clean, running water in the last 48 hours		
No	1	4.5
Yes	21	95.5
Soap is available at each hand hygiene station		
No	2	9.1
Yes	20	90.9
Single-use towels available at each hand hygiene station		
No	16	72.7
Yes	6	27.3
Management is committed to providing alcohol-based hand rubs and soaps at all times in the facility		
No	1	4.5
Yes	21	95.5

Table *2*: Training and Education

**Training and Education**	**Frequency**	**Per cent %**
Frequency of training received by HCW on hand hygiene each year		
At least once	8	36.4
Regular training for medical and nursing staff or all professional categories (at least annually)	8	36.4
Mandatory training for all professional categories at the commencement of employment, then ongoing regular training (at least annually)	6	27.3
Processes in place to confirm HCWs complete training on hand hygiene		
No	10	45.5
Yes	12	54.5
The document on the “5 Moments of Hand Hygiene in Healthcare” is easily available to all healthcare workers in the facility		
No	7	31.8
Yes	15	68.2
The document on the “Steps in Hand Hygiene” is easily available to all healthcare workers in the facility		
No	1	4.5
Yes	21	95.5
The document on the “National IPC/WASH Guidelines/Policy” is easily available to all healthcare workers in the facility		
No	8	36.4
Yes	14	63.6
Availability of IPC/WASH focal person with adequate skills who serves as a trainer for hand hygiene educational programs active within the facility		
No	2	9.1
Yes	20	90.9
Systems are in place to check hand hygiene compliance among HCWs quarterly		

No	8	36.4
Yes	14	63.6
Availability of a dedicated budget for IPC training, including hand hygiene in the facility		
No	13	59.1
Yes	9	40.9

Table *3*: Evaluation and Feedback

**Evaluation and Feedback**	**Frequency**	**Per cent %**
Regular (at least annual) ward-based audits are undertaken to assess the availability of hand hygiene resources in the facility		
No	3	13.6
Yes	19	86.4
Healthcare worker knowledge is assessed on the “indications for hand hygiene” (at least annually) after educational sessions in the facility		
No	3	13.6
Yes	19	86.4
Healthcare worker knowledge is assessed on the “correct technique for hand hygiene” (at least annually) after educational sessions in the facility		
No	2	9.1
Yes	20	90.9
Consumption of alcohol-based hand rubs regularly monitored (at least every 3 months) in the facility		
No	9	40.9
Yes	13	59.1
Consumption of soap regularly monitored (at least every 3 months) in the facility		
No	7	31.8
Yes	15	68.2

Frequency of direct observation of hand hygiene compliance using the "5 Moments of hand hygiene" in the facility		
Never	3	13.6
Irregularly	7	31.8
Annually	3	13.6
Every 3 months or more often	9	40.9
Frequency of direct observation of hand hygiene compliance using the "National Guideline on steps of hand hygiene" in the facility		
Irregularly	10	45.5
Annually	2	9.1
Every 3 months or more often	10	45.5
Immediate coaching/mentorship is provided to front line healthcare workers during hand hygiene compliance assessment in the facility		
No	5	22.7
Yes	17	77.3
Dissemination of hand hygiene compliance information/feedback is provided to Healthcare workers in the facility		
No	4	18.2
Yes	18	81.8
Dissemination of hand hygiene compliance information/feedback is provided to facility leadership/management		
No	4	18.2
Yes	18	81.8

Table 4: Reminders in the Workplace

**Reminders in the Workplace**	**Frequency**	**Per cent %**
A poster explaining the indications for hand hygiene		
Not displayed	4	18.2
Displayed in some wards/treatment areas	3	13.6
Displayed in most wards/treatment areas	5	22.7

Displayed in all wards/treatment areas	10	45.5
A poster explaining the correct use of hand rub		
Not displayed	2	9.1
Displayed in some wards/treatment areas	4	18.2
Displayed in most wards/treatment areas	6	27.3
Displayed in all wards/treatment areas	10	45.5
A poster explaining the correct handwashing technique		
Displayed in some wards/treatment areas	2	9.1
Displayed in most wards/treatment areas	9	40.9
Displayed at every sink in all wards/treatment areas	11	50
Frequency of systematic audit of all posters of evidence of damage occurs, with replacement in the facility		
Never	5	22.7
At least annually	14	63.6
Every 2-3 months	3	13.6
Hand hygiene promotion is undertaken by displaying and regularly updating posters		
No	14	63.6
Yes	8	36.4
Availability of hand hygiene information (e.g. leaflets) on the wards		
No	19	86.4
Yes	3	13.6
Availability of other workplace reminders (e.g. hand hygiene campaign screen savers, badges, stickers, etc.) throughout the facility		
No	19	86.4
Yes	3	13.6

Table *5*: Institutional Safety Climate for Hand Hygiene

Institutional Safety Climate for Hand Hygiene	Frequency	Per cent (%)
Availability of IPC/WASH committee in the health facility		
No	6	27.3
Yes	16	72.7
IPC/WASH committee meet regularly (at least monthly)		
No	9	56.3
Yes	7	43.7
IPC/WASH committee has a dedicated time to conduct active hand hygiene promotion		
No	8	50.0
Yes	8	50.0
Commitment of facility Health Service Administrator to support hand hygiene improvement		
No	5	22.7
Yes	17	77.3
Commitment of facility Medical superintendent to support hand hygiene improvement		
No	2	9.1
Yes	20	90.9
A commitment of facility DDNS/Matron to support hand hygiene improvement		
No	2	9.1
Yes	20	90.9
The facility has celebrated the Global Hand Hygiene day on the 5th of May for the last two years		
No	21	95.5
Yes	1	4.5
Systems available for designation of hand hygiene champions in the facility		
No	17	77.3

Yes	5	22.7
Systems available for recognition and utilization of hand hygiene role models in the facility		
No	20	90.9
Yes	2	9.1
Patients are informed about the importance of hand hygiene		
No	1	4.5
Yes	21	95.5
Initiatives to support hand hygiene e-learning are applied in the facility		
No	19	86.4
Yes	3	13.6
Hand hygiene target set to be achieved in the facility this year		
No	14	63.6
Yes	8	36.4
Systems available for intra-institutional sharing of reliable and tested local innovations		
No	18	81.8
Yes	4	18.2
Communications that regularly mentioned hand hygiene in the facility		
No	7	31.8
Yes	15	68.2
IPC/Hand hygiene included in staff appraisal in the facility		
No	9	40.9
Yes	13	59.1
New employees/students/intends are provided orientation on IPC/Hand hygiene in		
No	2	9.1
Yes	20	90.9
